# Impact of Concurrent Anticonvulsant Use on Seizure Parameters and Clinical Outcomes of Electroconvulsive Therapy in Bipolar Disorder: A Systematic Review and Meta-Analysis

**DOI:** 10.1192/j.eurpsy.2025.435

**Published:** 2025-08-26

**Authors:** I. Borja De Oliveira, E. Tolotti Leite, I. Santos Raposo Andrade, M. L. Geremias, A. L. Stephany, A. V. de Vasconcelos, D. Xavier, F. Wagner, G. A. M. Alves, M. O. Pozzolo Pedro, D. Soler Lopes, A. L. Balduino de Souza, M. C. Carbajal Tamez, J. Quevedo

**Affiliations:** 1 School of Medicine, University of São Paulo, São Paulo; 2 Municipal Health Department of Nova Andradina, Nova Andradina; 3 Pontifical Catholic University of Campinas, Campinas; 4 University of the Joinville Region, Joinville; 5 Integrated Medicine Service, Jacareí; 6 Afya College of Medical Sciences of Santa Inês, Santa Inês, Brazil; 7 ECPE - PPCR Program, Harvard T. H. Chan School of Public Health, Boston, United States; 8 School of Medicine, Pontifical Catholic University of Rio Grande do Sul, Porto Alegre, Brazil; 9 Humanitas University, Milan, Italy; 10 Departament and Institute of Psychiatry, University of São Paulo, São Paulo, Brazil; 11 Lancaster University, Lancaster, United Kingdom; 12 Evangelical University of Goias, Anapolis, Brazil; 13 Department of Psychiatry and Behavioral Sciences at McGovern Medical School; 14 Center for Interventional Psychiatry, Faillace Department of Psychiatry and Behavioral Sciences, McGovern Medical School, The University of Texas Health Science Center at Houston, Houston, United States

## Abstract

**Introduction:**

Treatment for bipolar disorder (BD) predominantly focuses on psychopharmacology, including lithium, antipsychotics, and anticonvulsants. Electroconvulsive therapy (ECT) is highly effective for managing manic or depressive episodes, yet studies on the effects of anticonvulsant therapy as a modifying factor of clinical outcome during ECT are scarce.

**Objectives:**

To evaluate how concurrent anticonvulsant use affects seizure parameters and clinical outcomes of ECT in BD patients.

**Methods:**

A comprehensive search of multiple databases (MEDLINE, Embase, Web of Science, PsycINFO, Cochrane Central Register of Controlled Trials, World Health Organization International Clinical Trials Registry Platform, ClinicalTrials.gov) was conducted on October 2, 2024, without language or publication date restrictions. Eligible studies included clinical trials and retrospective analyses comparing BD patients undergoing ECT with and without anticonvulsant use. Random-effects models were applied for a sufficient number of studies, while fixed-effects models were used for fewer studies. Subgroup and sensitivity analyses were conducted.

**Results:**

Six studies met the criteria, involving 359 participants (mean age: 29.7 years; 31.2% female). Five studies focused on the effect of concomitant treatment with valproate during a manic episode, and only one study included subjects in treatment with other anticonvulsants during different mood episodes of BD. Anticonvulsant users required significantly higher minimal electrical dosages to achieve adequate seizures (SMD = 0.71, 95% CI [0.46 to 0.95], p < 0.0001), as indicated by higher seizure thresholds and stimulus doses. Additionally, anticonvulsant use was associated with a significantly shorter seizure duration (SMD = -0.75, 95% CI [-1.10 to -0.41], p < 0.0001). However, no significant differences in symptomatic improvement were found between those using and not using anticonvulsants (SMD = 0.03, 95% CI [-0.19 to 0.25], p = 0.78).

**Conclusions:**

Concurrent anticonvulsant use in BD patients undergoing ECT is associated with higher seizure thresholds and shorter seizure durations, but this does not affect clinical outcomes regarding disease severity. Based on these findings, discontinuation of anticonvulsants during ECT may not be necessary. This review was limited by the small number of studies, small sample sizes, and considerable heterogeneity. Additionally, the majority of the studies analyzed only included patients in the manic state of the illness. Further research is needed to explore whether variations in seizure parameters are linked to individual clinical outcomes in BD patients, the impact of different anticonvulsants on these parameters and the outcome for depressive and mixed episodes of bipolar disorder.

**Disclosure of Interest:**

I. Borja De Oliveira: None Declared, E. Tolotti Leite: None Declared, I. Santos Raposo Andrade: None Declared, M. Geremias: None Declared, A. Stephany: None Declared, A. de Vasconcelos: None Declared, D. Xavier: None Declared, F. Wagner: None Declared, G. A. M. Alves: None Declared, M. O. Pozzolo Pedro: None Declared, D. Soler Lopes: None Declared, A. Balduino de Souza: None Declared, M. Carbajal Tamez: None Declared, J. Quevedo Shareolder of: Instituto de Neurociencias Dr. Joao Quevedo, Grant / Research support from: LivaNova; and receives copyrights from Artmed Editora, Artmed Panamericana, and Elsevier/Academic Press, Consultant of: EMS, Libbs, and Eurofarma, Speakers bureau of: Myriad Neuroscience and AbbVie.

